# Children’s physical activity and parents’ perception of the neighborhood environment: neighborhood impact on kids study

**DOI:** 10.1186/1479-5868-10-39

**Published:** 2013-03-27

**Authors:** Karyn A Tappe, Karen Glanz, James F Sallis, Chuan Zhou, Brian E Saelens

**Affiliations:** 1 Department of Biostatistics and Epidemiology, Perelman School of Medicine, University of Pennsylvania, Philadelphia, USA; 2 Current address: Department of Psychology, Rowan University, Glassboro, NJ, USA; 3 Department of Biostatistics and Epidemiology, Perelman School of Medicine and School of Nursing, University of Pennsylvania, Philadelphia, USA; 4 Department of Psychology, San Diego State University, San Diego, CA, USA; 5 Current address: Department of Family and Preventive Medicine, University of California, San Diego, CA, USA; 6 Seattle Children’s Research Institute, P.O. Box 5371, Seattle, WA 98145, USA; 7 Department of Pediatrics, School of Medicine, University of Washington, Seattle, USA

**Keywords:** Built environment, Perceptions, Recreation, Play

## Abstract

**Background:**

Physical activity is important to children’s physical health and well-being. Many factors contribute to children’s physical activity, and the built environment has garnered considerable interest recently, as many young children spend much of their time in and around their immediate neighborhood. Few studies have identified correlates of children’s activity in specific locations. This study examined associations between parent report of their home neighborhood environment and children’s overall and location-specific physical activity.

**Methods:**

Parents and children ages 6 to 11 (n=724), living in neighborhoods identified through objective built environment factors as high or low in physical activity environments, were recruited from Seattle and San Diego metropolitan areas, 2007–2009. Parents completed a survey about their child’s activity and perceptions of home neighborhood environmental attributes. Children wore an accelerometer for 7 days. Multivariate regression models explored perceived environment correlates of parent-reported child’s recreational physical activity in their neighborhood, in parks, and in general, as well as accelerometry-based moderate-to-vigorous activity (MVPA) minutes.

**Results:**

Parent-reported proximity to play areas correlated positively with both accelerometery MVPA and parent-reported total child physical activity. Lower street connectivity and higher neighborhood aesthetics correlated with higher reported child activity in the neighborhood, while reported safety from crime and walk and cycle facilities correlated positively with reported child activity in public recreation spaces.

**Conclusions:**

Different aspects of parent’s perceptions of the neighborhood environment appear to correlate with different aspects of children’s activity. However, prioritizing closer proximity to safe play areas may best improve children’s physical activity and, in turn, reduce their risk of obesity and associated chronic diseases.

## Background

Neighborhood environments may play an important role in children’s planned and incidental physical activity [1,2]. Neighborhood built environment comprises buildings, roads, open spaces, and sidewalks [3] and provide opportunities or barriers to physical activity [4]. The neighborhood social environment, as it relates to physical activity, includes personal safety from crime and traffic [4]. Because children have less autonomy than adults, these neighborhood environmental characteristics, as well as their parents’ perceptions of these characteristics, may have particular impact on their physical activity [5,6]. However, in a recent systematic review, only 30% of associations between parent perceptions of the environment and children’s activities was significant in the anticipated direction. The most consistently supported predictor of children’s reported physical activity was pedestrian safety structures (e.g., crosswalks). None of the environmental attributes were consistently related to children’s objectively-measured physical activity [2].

One possible reason for inconsistencies is that few studies have attempted to relate environmental attributes to children’s activity in specific locations. Neighborhood attributes may be more related to physical activity in specific locations in the neighborhood, whereas overall physical activity may be influenced by a broader range of neighborhood, school, community, family, and other factors. Active transport has been the topic most frequently studied in relation to location of activity (e.g., in the neighborhood [7-9]). Other research has only considered inside versus outside play [10] and activity in school play areas [11]. More detailed studies of environmental correlates of children's physical activity in specific locations may be informative, particularly for public policy and urban planning projects that target specific transit and recreation-related infrastructure.

The present study examines associations between parent reports of their neighborhood environment and children’s activity within the neighborhood and in parks. Because measurement modality for physical activity influences findings [2], the present study examined both parent-reported and accelerometer-assessed children’s physical activity. The parent reports herein emphasize children’s recreational activity.

## Methods

### Context

The analyses presented here are based on the baseline data of the Neighborhood Impact on Kids (NIK) Study, an NIH-funded longitudinal, observational cohort study of children and their parents in Seattle/King County, WA and San Diego County, CA. NIK was designed to evaluate the association of neighborhood environment factors with children and parent’s weight status and weight-related behaviors, including physical activity [12]. This study was approved by the Institutional Review Boards at Seattle Children’s Hospital and San Diego State University.

### Participants

Participants were selected using a two-stage stratified cluster sampling. In the first stage, census blocks (hereafter, “neighborhoods”) were selected based on their physical activity opportunities, categorized as having high versus low physical activity opportunities based on Geographic Information Systems (GIS)-generated measures. In the second stage, households within the identified neighborhoods were contacted using information from a commercial marketing firm. Participants consisting of one child aged 6 to 11 and one parent/caregiver per household were recruited. Children and parents were required to live in one of the identifıed neighborhoods; be able to engage in at least moderate-intensity physical activity; not have underlying medical conditions associated with obesity (e.g., Cushing’s syndrome) or be actively involved in medical treatment that has substantive impact on growth (e.g., growth hormone treatment). Further details on both neighborhood and individual inclusion/exclusion can be found elsewhere [12]. The eligible block groups have been analyzed and found similar to the county as a whole [12].

### Recruitment and response rates

Participants were recruited between September 2007 and January 2009. A total of 8,616 recruitment letters were sent, and 4,975 households were contacted by phone to explain the study and assess eligibility and willingness to participate. In San Diego County, 366 families consented to participate, and in Seattle/King County, 372 consented (14.6% consent rate). A total of 730 families from both sites completed their initial visit.

### Procedures

At an office or home visit, parents provided consent and children provided assent. Parents and children had their height, weight and waist circumference measured. They were then provided with an accelerometer and instructed on how to have the child wear it. The parent was asked to complete a 25-page survey (online or on paper) that included questions about the neighborhood and their child’s physical activities. The study staff called participants once during the week following the visit to answer any questions and encourage daily wearing of the accelerometer.

### Measures

#### Accelerometer-measured children’s physical activity

Child physical activity was objectively measured by the GT1M Actigraph^®^ accelerometer [13]. Children were asked to wear the accelerometer for seven consecutive days, at least 10 hours per day during their waking hours. The accelerometry data were downloaded and screened for completeness and possible irregularities/malfunction upon return by mail. A valid wearing/waking hour included hours without any instances of 20 minutes of consecutive zero activity count epochs. Children were asked to wear the accelerometer again if <6 days of valid data (minimum of 10 hours per day) were recorded (102 children, or 13.8% of sample).

We calculated average minutes of moderate-to-vigorous physical activity (MVPA) per day using age-based 3-MET cut-points [14] and the Evenson MVPA calculation method [15], as they can produce quite different results and the definitive cut-point for children has not been established.

#### Neighborhood environment survey

Table 1 provides descriptions of survey sections analyzed in the present study (see [16] for the full questionnaire). Neighborhood environment perception items were taken from previously-evaluated measures [17-19]. Most of the survey subscales have shown good internal consistency and test-retest reliability [17,19,20]. Items addressed various aspects of the built and social environments, such as proximity to destinations, inaccessibility of recreation facilities, presence of suitable play areas, street connectivity, aesthetics, traffic safety, and crime safety [21]. The internal consistency of the subscales used in the present study was confirmed using Cronbach’s alpha.

**Table 1:** Subscales on the NIK Self-Report Survey used in the present report, with descriptions, scoring, and internal consistency, Seattle and San Diego, 2007-2009

**Name**	**# Items**	**Example items, response options, and scale development**	**Alpha**	**Mean (S.D.) or proportions**
Predictor variables*				
Getting Around in Your Neighborhood[19]	22 (5 subscales)	Subjective evaluation of ease or difficulty of traveling in neighborhood due to various issues: “Parking is difficult; …There are sidewalks on most streets; … There are trees along streets.” Each item scored on 4-point Likert from 1 (Strongly Disagree) to 4 (Strongly Agree). Subscales: i) street connectivity; ii) walking/cycling facilities; iii) neighborhood aesthetics; iv) traffic safety; v) safety against crime. Subscales adapted from previous research [19]	Scale i: .42	2.76 (0.76)
			Scale ii: .70	2.53 (0.88)
			Scale iii: .81	3.03 (0.66)
			Scale iv: .60	2.37 (0.51)
			Scale v: .82	2.07 (0.66)
Proximity to Locations[19]	25 (2 subscales)	“About how long would it take you to walk from your home to the nearest places listed?” Scale: from 1 (1–5 minutes) to 5 (31+ minutes) or 6 (Don’t know). Reverse recoded per [21] to indicate proximity to locations. Subscales: i) Stores/services and ii) Recreation areas (swimming pool; indoor recreation facility; water recreation areas; trails; basketball court; other fields/courts; parks; playgrounds; schools with available facilities).	Scale i: .92	2.67 (0.92)
			Scale ii: .84	2.55 (0.82)
Barriers to Walking and Biking[17]	14 (2 subscales)	“It is difficult for my child to walk or bike to the closest park or playground because… there are no sidewalks; …the route is boring; …my child has too much stuff to carry.” Scale: 4-point Likert from 1 (Strongly Disagree) to 4 (Strongly Agree).Subscales: i) Logistics ii) Route characteristics.	Scale i: .77	1.62 (0.55)
			Scale ii: .79	1.91 (0.70)
Barriers to Activity in Your Neighborhood[17]	9 (2 subscales)	“It is difficult for my child to be active in the local park or the streets/neighborhood near our home because… there is no choice of activities; …there is no equipment; …it is not safe because of traffic.” Scale: 4-point Likert from 1 (Strongly Disagree) to 4 (Strongly Agree).Subscales: i) Perceived lack of appropriate play areas; ii) Crime activity.	Scale i: .81	1.81 (0.67)
			Scale ii: .72	1.57 (0.73)
Outcome Measures**				
Neighborhood activity[17]	4	“How often is your child physically active: In your driveway or alley? …In a local street, sidewalk, or vacant lot?” Options: 1 (Never) to 6 (4 days/week or more). Recoded to indicate number of times per month, and numeric responses summed.The total was dichotomized at 4 days per week (16 days per month) to indicate physical activity in neighborhood.	.76	NA
Park activity[17]	4	“How often is your child physically active in/at the following locations: Trails/paths? …Small public park? …Large public park? …Open space?” Options: 1 (Never) to 6 (4 times/week or more). All 4 items recoded, summed and dichotomized at 2+ days per week to indicate any activity in parks/trails/open areas.	.67	NA
60+ minute activity days (outside of school)[17]	2	“How many days is/was your child physical activity for a total of at least 60 minutes per day (do not include school based activities)?” (Scored: 0–7 days). Two items: i) the past seven days; ii) Over a typical week. Averaged and then dichotomized at 5 days/week.	.93	NA

NA = Not applicable due to dichotomous nature of variable.

* Note: All predictor variables were directionally coded so that a higher number indicated an environment more conducive to being physically active.

** See [16] for full questionnaire set.

#### Physical activity survey

Parents completed three survey sections about their children’s physical activity (see Table 1) [17]. First, frequency of children’s physical activity was assessed in their driveway/alley, neighborhood yards or driveways, streets or sidewalks, and in a nearby cul-de-sac or dead-end street (hereafter grouped into “neighborhood activity”). Second, activity in parks and park-like environments (hereafter grouped into “park activity”) was assessed through four questions inquiring about frequency of physical activity on paths/trails, at small public parks/playgrounds, at large public parks, or in open spaces.

For each category of activity (neighborhood activity or park activity), the ordinal responses were rescaled and summed to indicate number of times per month active in any possible combination of the specific locations that made up neighborhood or park activity (ex. twice a week active in alley plus twice a week active in driveway), and then dichotomized at 1) four times per week (16 times per month) or more (yes or no) for neighborhood activity and 2) two times per week or more (yes or no) for park activity. These scales were dichotomized because they were ordinal rather than interval scales, and thus not suitable for use as continuous variables. The cut points represent the approximate median value (rounded to nearest whole day) for each one of these variables, as no more theoretically-justified cut point has been established for such location-specific activity measurement.

In the third survey section, parent-reported days per week that their child performed 60 or more minutes of physical activity was assessed using two items that were averaged [22]. This average was dichotomized at 5 or more days per week of activity, the median value reported. It is referred to hereafter as “60+ minute activity days.”

#### Physical activity environment

Study census blocks (“neighborhoods”) were selected based on a multi-dimensional assessment of physical activity opportunities generated from parcel and street network data in a GIS to evaluate within each region each neighborhood’s (a) built environment related to walkability (weighted sum of z-scores by region for residential density, intersection density, land use mix, retail floor area ratio) [23] and (b) proximity of a "high quality" park. "High physical activity neighborhoods” were those with an above median walkability index score and at least one park within the neighborhood or the ¼ mile buffer around the neighborhood that scored high on a park quality audit (Environmental Assessment of Public Recreation Spaces) [16,24]. "Low physical activity neighborhoods" were below these thresholds (see [12]). This dichotomous variable was included in all multivariate models.

#### Household demographics

Demographics were included as covariates in the multivariate models: child sex, age, race (white or non-white due to low numbers of most non-African American minority groups), BMI percentile, ethnicity (Hispanic or not), and household income (<$50,000/yr, $50-$100,000/yr and $100,000+/yr).

#### Analyses

Linear and logistic bivariate models were calculated for continuous and dichotomous outcomes respectively using single predictor regression models. Then, the three dichotomized outcome measures (neighborhood activity at 4 times or more per week [yes/no], park activity at 2 times or more per week [yes/no], and 60+ minutes of physical activity at 5 days per week [yes/no]) were analyzed with multivariate logistic regression models. Two groups of predictors were considered: demographics and neighborhood environment factors. Given the large number of potential predictors, a manual, backwards stepwise regression approach was used, starting with an initial model of all possible predictors (“full model”) and manually dropping the least significant term one at a time, then comparing the reduced model to the previous model using the likelihood ratio test. If the model fit was significantly worse, the removed term was returned and the next least significant term examined, until no further term could be removed without significant reduction in model goodness of fit. Therefore, non-significant covariates that stabilized the model fit were retained. The likelihood ratio test for this “final” model relative to the null model (intercept only) is reported.

Average MVPA minutes per day by accelerometer was treated as a continuous outcome and analyzed with multiple linear regression; skewness and kurtosis were compared against existing standards [25], and indicated no need for transformation of the outcome variable. The final models (one based on 3+ MET cut points and one based on Evenson cut points) were identified by a similar manually-performed backwards stepwise procedure as for the logistic regression models.

Because participants were recruited from particular neighborhoods that met our inclusion criteria, we evaluated the data for clustering effects that might necessitate multi-level modeling. The families were recruited from a large number of neighborhoods (n=310) that are not necessarily geographically close. The numbers of families from the same neighborhood tend to be small (mean: 2.35, range 1–16); 81% of the neighborhoods had three or fewer participating families (38% had only one family). Therefore we did not expect a clustering effect among the families. The intra-class correlation (ICC) for MVPA by neighborhood was only .06. As a result, multi-level modeling was not deemed appropriate and we report results based on participant-level, fixed effects models only.

All statistical analyses were performed using the SPSS statistical software version 19.0 (IBM, Armonk, NY). P < .05 was considered statistically significant.

## Results

### Participant characteristics

The average age of children in the study was 9.14 ± 1.56; 81.6% were white and 16.5% reported themselves as Latino. Sex distribution was equal (49.3% female). The average BMI percentile score of the children was 62.24 ± 27.16; 15.5% were classified as overweight (85-94% BMI percentile) and 14.4% as obese (95% + BMI percentile). Family income was reported above $100,000 per annum by 49.4% and below $50,000 for 13.6% of families.

Children spent an average of 146.0 minutes (SD=53.4) based on the 3+ METS cut points and 46.7 minutes (SD=21.4) based on the Evenson cut point in MVPA per day based on the accelerometer, suggesting a predominance of low-moderate physical activity not captured by the higher Evenson cut points.

### Physical activity and reported environment

#### Parent-reported outcomes: neighborhood physical activity

Table 2 shows the univariate, full, and final multivariate models on parent-reported child neighborhood activity. Four neighborhood environment and three demographic variables were in the final model (χ^2^ = 41.04, p *<* .001), four of which were statistically significant. Girls were less likely to be active than boys (OR = 0.68). Lower street connectivity (OR = 0.77) and better aesthetics (OR = 1.57) increased the odds of being active at least 4 days a week in the neighborhood, while perceived lack of suitable play areas lowered the odds of being active (OR = 0.74).

**Table 2:** Logistic regression: odds of 4+ days of parent-reported children’s neighborhood activity as predicted by parent-reported demographics and neighborhood characteristics

	**Univariate model**		**Full model**		**Final model**	
	**OR**	***P***	**OR**	***P***	**OR [95% CI]**	***P***
**Demographics (reference category)**						
Child race(White)	1.06	.75	1.19	.41		
Child age	0.92	.09	0.91	.08	0.91 [0.82 – 1.01]	.08
Child sex (M)	0.73	.04	0.67	.01	0.68 [0.49 – 0.93]	.02
Hispanic (N)	0.81	.30	0.85	.48		
Child’s BMI %	1.00	.69	1.00	.41		
Household income < $50,000 (N)	0.76	.21	0.90	.70	0.87 [0.54 – 1.41]	.57
Household income $50-$100,000 (N)	0.87	.36	0.90	.56	0.91 [0.65 – 1.30]	.62
**Neighborhood environment**						
Physical activity environment (GIS)	0.83	.23	0.79	.20	0.84 [0.60 – 1.16]	.29
Safety from crime	1.17	.17	0.90	.47		
Traffic safety	1.77	<.001	1.46	.06		
Street connectivity	0.82	.05	0.76	.02	0.77 [0.62 – 0.96]	.03
Neighborhood aesthetics	1.68	<.001	1.47	.01	1.57 [1.21 – 2.03]	<.01
Walk/cycle facilities	1.05	.60	0.99	.94		
Proximity to stores	1.00	.96	0.94	.63		
Proximity to play areas	1.19	.07	1.17	.29		
Barriers to walking/biking: Logistics	0.68	<.01	0.85	.40		
Barriers to walking/biking: Route	0.75	<.01	1.11	.55		
Barriers to activity: Perceived lack of appropriate play areas	0.64	<.001	0.79	.12	0.74 [0.58 – 0.95]	.02
Barriers to activity: Crime	0.81	.04	1.10	.48		
Constant			2.00	.54	4.35	.05

N = 673, Seattle and San Diego, 2007–2009.

Percent of cases correctly classified with this model: 63.5%.

*OR *Odds ratio.

*GIS *Geographic Information Systems data.

#### Parent-reported outcomes: physical activity in park areas

As seen in Table 3, the final model for reported park activity comprised seven neighborhood environment and three demographic variables (χ^2^ = 64.87, p *<* .001), six of which were statistically significant. Being Hispanic (OR = 1.56), better reported safety from crime (OR = 1.30), higher neighborhood aesthetics (OR = 1.38), better walking/cycling facilities (OR = 1.40), and closer proximity to play areas (OR = 1.22) were significantly associated with increased odds of reported park activity on at least 2 days per week. Reported lack of appropriate play areas was significantly associated with lower odds of reported park activity (OR = 0.74).

**Table 3:** Logistic regression: odds of 2+ days of parent-reported children’s park activity as explained by parent-reported demographics and neighborhood environment

	**Univariate model**		**Full model**		**Final model**	
	**OR**	***P***	**OR**	***P***	**OR [95% CI]**	***P***
**Demographics (reference category)**						
Race (White)	0.88	.50	0.89	.59		
Age	0.99	.79	0.97	.61		
Sex (M)	1.05	.73	0.94	.71		
Hispanic (N)	1.49	.05	1.57	.06	1.56 [0.99 – 2.45]	.05
Child’s BMI percentile	1.00	.15	1.00	.20		
Household income < $50,000 (N)	1.14	.55	1.30	.34	1.17 [0.70 – 1.98]	.55
Household income $50-$100,000 (N)	1.08	.64	1.21	.30	1.16 [0.82 – 1.65]	.41
**Neighborhood environment**						
Physical activity environment (GIS)	1.34	.05	1.08	.66	1.09 [0.78 – 1.53]	.61
Safety against crime	1.35	.01	1.23	.14	1.30 [1.00 – 1.67]	.05
Street connectivity	1.08	.42	0.91	.43		
Neighborhood aesthetics	1.82	<.001	1.39	.02	1.38 [1.06 – 1.80]	.02
Traffic safety	1.87	<.001	0.93	.72		
Walk and cycle facilities	1.59	<.001	1.49	<.001	1.40 [1.16 – 1.71]	.001
Proximity to stores	1.31	.001	1.01	.94		
Proximity to play areas	1.55	<.001	1.24	.13	1.22 [0.98 – 1.53]	.07
Barriers to walking/ cycling: logistics	0.65	.002	0.84	.36		
Barriers to walking/ cycling: route	0.59	<.001	1.08	.67		
Barriers to activity: perceived lack of appropriate play areas	0.58	<.001	0.80	.15	.74 [0.57 – 0.96]	.03
Barriers to activity: crime	0.73	.003	0.86	.25		
Constant			0.31	.31	.09	.002

N = 676, Seattle and San Diego, 2007–2009.

Percent of cases correctly classified with this model: 63.5.

*OR *Odds ratio.

*GIS *Geographic Information Systems data.

#### Parent-reported outcomes: 60+ minute activity days

The final model for reportedly engaging in 60+ activity minutes at least 5 days per week contained three neighborhood environment variables and four demographic variables (χ^2^ = 34.41, p *<* .001 (see Table 4), three of which were statistically significant. Reported closer proximity to play areas (OR = 1.29) was the only environment variable significantly associated with greater odds of children’s 60+ activity minutes per day 5+ days per week. Child age and sex also were significant correlates such that girls (OR = 0.57) and older children (OR = 0.88) were less likely to reportedly engage in this level of activity.

**Table 4:** Logistic regression: odds of 5+ days per week of parent-reported overall child activity as explained by parent-reported demographics and neighborhood environment

	**Univariate model**		**Full model**		**Final model**	
	**OR**	***P***	**OR**	***P***	**OR [95% CI]**	***P***
**Demographics (reference category)**						
Child Race (White)	0.94	.76	.98	.94		
Child Age	0.88	.01	.89	.02	0.88 [0.80 – 0.98]	.02
Child Sex (M)	0.60	.001	.54	<.01	0.57 [0.41 – 0.77]	<.01
Hispanic (N)	0.76	.19	.83	.43	0.83 [0.53 – 1.28]	.40
Child’s BMI percentile	0.99	.69	1.00	.40		
Household income < $50,000 (N)	0.81	.33	.93	.80	0.83 [0.50 – 1.38]	.47
Household income $50-100,000 (N)	0.83	.22	.86	.40	0.83 [0.59 – 1.17]	.28
**Neighborhood environment**						
Physical activity environment (GIS)	1.14	.39	1.11	.56	1.04 [0.75 – 1.44]	.81
Safety against crime	1.05	.65	.84	.23		
Street connectivity	1.00	.99	1.02	.85		
Neighborhood aesthetics	1.33	.01	1.21	.16		
Traffic safety	1.49	.01	1.48	.05	1.31 [ 0.96 – 1.80]	.09
Walk/cycle facilities	0.98	.82	.89	.28		
Proximity to stores	1.08	.32	.90	.41		
Proximity to play areas	1.33	.002	1.46	.01	1.29 [1.05 – 1.60]	.02
Barriers to walking/cycling: logistics	0.81	.14	.96	.83		
Barriers to walking/cycling: route	0.85	.85	1.22	.26		
Barriers to activity: perceived lack of appropriate play areas	0.79	.04	1.00	.99		
Barriers to activity: crime	0.82	.06	.89	.39		
Constant			.78	.82	0.99	.98

Percent of cases correctly classified with this model: 58.5.

*OR *Odds Ratio.

*GIS *Geographic Information Systems data. Note: For ease of discussion in the text, the reciprocal of the odds ratio (1/OR) is sometimes reported.

(N = 675).

#### Accelerometry-measured MVPA minutes

The 3-MET cut point was used for the main analysis, and the multivariate linear model explained 49% of the variance in accelerometer-based MVPA (F_3,710_ = 227.88, p *<* .001) (see Table 5), with only one neighborhood and two demographic factor retained in the model. Accelerometry MVPA minutes was positively related to greater reported proximity to play areas (B = 4.12, p < .05). Younger age was associated with higher MVPA minutes (B = -22.44, p *<* .001) and males were more active than females (B = -25.64, p *<* .001). Use of the Evenson cut point (F_6, 687_ = 23.24, p < .001) explained 17% of the variance in MVPA and identified two additional significant correlates: lower BMI percentile (B = -0.08, p < .05) and higher household income (using two dummy variables, ps < .05) related to more physical activity.

**Table 5:** Linear regression: pediction of accelerometry-based minutes of MVPA per day from parent-reported demographics and neighborhood environment

**Model**	**Univariate models for 3 MET cut point**	**Full model for 3 MET cut point**	**Final model for 3+ METs**	**Final model using Evenson cut points**
	**B**	**B**	**B [95% CI]**	**B [95% CI]**
**Demographics**				
Child Race (White)	-3.25	1.39		
Child Age	-22.38**	-22.60**	-22.44** [-24.24 – -20.64]	-2.57** [-3.51 – -1.63]
Child Sex (F)	-29.40**	-26.80**	-25.64** [-31.26 – -20.01]	-14.23** [-17.19 – -11.27]
Hispanic (Y)	-5.34	-0.93		
Child’s BMI percentile	0.03	-0.06		-0.08* [-0.13 – -0.03]
Household income < $50,000 (Y)	-8.73	-6.64		-4.97* [-9.49 – -0.45]
Household income $50-100,000 (Y)	-3.60	-3.09		-4.24* [-7.44 – -1.04]
**Neighborhood Environment**				
Physical activity environment (GIS)	2.94	3.52		
Safety against crime	-0.11	3.70		
Street connectivity	0.13	-0.74		
Neighborhood aesthetics	0.11	0.64		
Traffic safety	1.93	-1.38		
Walk/cycle facilities	-2.43	-1.29		
Proximity to stores	2.74	1.33		
Proximity to play areas	3.54	2.25	4.12* [0.66 – 7.58]	2.12* [0.30 – 3.95]
Barriers to walking/cycling: logistics	-2.17	-2.10		
Barriers to walking/cycling: route factors	-1.08	2.55		
Barriers to activity: perceived lack of appropriate play areas	-5.35	-3.15		
Barriers to activity: crime	-1.27	2.85		
Constant		357.08**	353.85 [334.88 – 372.83]	79.07 [68.22 – 89.93]

All values expressed as unstandardized B.

GIS = Geographic Information Systems data.

* *P <*.05.

** *P <*.01.

(N = 711).

## Discussion

Several reported neighborhood environment correlates were found for child physical activity in the neighborhood, supporting the idea that stronger correlates will be identified for behaviors if the contexts of the correlates and behaviors are aligned [2,26,27]. Higher probability of children being reported active in the neighborhood was related to lower reported street connectivity (e.g., more cul-de-sacs), as found in another recent study [28]. Low connectivity reduces traffic volumes, providing safer neighborhood places to play. This finding contrasts with higher rates of transport-based physical activity among adults and children living in neighborhoods with higher street connectivity, such as more child active travel to/from school, particularly in low traffic areas [29]. Because adults are not likely to play in the cul-de-sacs and alleys like their children, these findings seem to present a tradeoff between child activity and adult activity based on physical environment. These findings together highlight the importance of examining specific types of physical activity (and subgroups within the population such as children versus adults) in relation to the environments in which the activity does or could occur, to know where best to target limited resources for public policy or urban planning projects. Creative planning solutions could allow parents and families to engage in active transportation to work, school, and other destinations allowed by better street connectivity while providing opportunities for children’s unstructured play in the neighborhood either in streets (e.g., traffic calming strategies) or by providing other proximal spaces for active play (e.g., more playgrounds).

As with adults’ recreational physical activity [30], we found that aesthetics were related to more child neighborhood physical activity. Favorable aesthetics may improve the enjoyment of being active in neighborhoods. However, Limstrand, 2008 [31] summarized the limited literature on this topic and found that two out of three studies reported no association between aesthetics and children’s physical activity level. Another review found little relation between vegetation and children’s reported activity [2]. Attractive buildings and gardens may have provided a sense of order for our particular sample of parents, making them more comfortable to let their children play and be active outside.

More frequent children’s park activity was associated with better safety from crime, aesthetics, walking/cycling facilities, and suitable play area availability. Prior evidence highlights the importance to children’s physical activity of having better accessibility to play areas [19,32]. Regarding aesthetics, we found previously that neighborhood aesthetics were perceived as higher by parents of children with more frequent park-based physical activity [19]. This prior study however found no relation between neighborhood safety from crime and park activity. Differences in sample characteristics and the types of urban areas and neighborhood environments may explain this discrepancy.

Proximity to play areas was the only correlate of total physical activity in the present study, and it applied to parent-reported child overall activity as well as to child accelerometer-based MVPA (both scoring methods). Others have found that play area proximity is consistently related to physical activity (as reviewed in [2]). Perhaps this association with overall physical activity reflects the fact that, for children, such physical activity is more discretionary than other physical activity in which children engage (e.g., school-based physical activity) or that higher levels of physical activity are only generally reached by children routinely using such play areas. Having few significant environmental correlates of the overall measures of physical activity is a finding consistent with previous literature [2]. Environmental influences are expected to be specific to domain of physical activity, such as transport or recreation [26,33], or physical specific location, such as neighborhood or park [5]. Thus, total physical activity measures may underestimate the importance of individual neighborhood environmental factors, although are useful given the association with health outcomes. Measuring physical activity in specific contexts will provide more useful information to those agencies and programs attempting to institute environmental changes to increase activity in these contexts.

Notably though, access or proximity to recreation areas was related to all physical activity outcomes in the present study, whether location-specific or total, parent-reported or objectively measured. This impressive consistency in a literature characterized by inconsistency [2] suggests that proximal play areas could be a powerful influence on child physical activity, and that this may be a useful focal point for cities that are developing more active-friendly neighborhoods. Recreational physical activity is the dominant domain of activity for children, and children typically have very low levels of physical activity while indoors [34], so it is reasonable that having places to play near the home would emerge as an important correlate of physical activity. Conversely, lack of accessible places to play could be a very strong barrier to children's activity.

Additional analyses are needed to examine whether objective or perceived built environment, or what combination of these, are more strongly related to children’s physical activity and where that physical activity occurs. The length of time spent being active in specific locations would also provide added value. A longitudinal study design would make causality inferences between the predictors and activity outcomes more feasible to determine, as would natural experiments in which children’s behavior is examined before and after environmental changes. The present study did not include objective location or environment data such as what can be measured using GPS and GIS, which would allow us to objectively measure actual distances to play locations relative to amount of activity, rather than relying on parental reports, some of which had low internal consistency.

Among study strengths, children from a diversity of neighborhoods were recruited from two distinct regions, and virtually all measures demonstrated good retest reliability. However, most children were from relatively affluent families, with representative but limited racial/ethnic diversity. The recruitment rate of this study was lower than in survey-only studies, likely due to the added burden of an office or in-home visit (to measure anthropometrics) and accelerometry, leading to self-selection bias of higher SES families. However, this higher SES does not seem to have resulted in abnormally high activity rates relative to a national sample [35,36]. Parental perception was a limiting factor in the assessment of the neighborhood environment, and measurements such as distances to parks and recreation areas were subjective and potentially prone to error. These analyses were limited to recreational activity and not transport activity, a potentially significant limitation given the substantial role that active transport by children to school can play in some neighborhoods and for their overall physical activity.

## Conclusion

The present study identified multiple conceptually-congruent neighborhood environment correlates of children's physical activity in neighborhoods and parks. Because these are the most common places for children to be active [9], and are also related to overall physical activity levels, these results have public health significance. If neighborhoods, parks, schools, private recreation facilities, and roadways can all be designed to optimize physical activity, then the cumulative effect could be expected to increase physical activity and reduce the risk of obesity and chronic diseases. Present results suggest that specific environmental changes may improve children's physical activity. However, these hypotheses need to be tested further. If confirmed, the evidence will provide justification and impetus for policy changes that will ensure more children live in environments that support active lifestyles. Proximity to play areas was related to all child physical activity outcomes in the present study, making this a high priority for further study and consideration of policy solutions to ensure all children have safe places to play near their homes.

## Competing interests

The authors have indicated they have no conflicts of interest or financial relationships relevant to this article to disclose.

## Authors’ contributions

JS, BS and KG conceived the overall NIK study. JS and BS collected the data. KG, BS, and KT conceptualized the manuscript. KT and CZ analyzed the data. All authors have participated in drafting or revising of the manuscript; and all have approved the manuscript as submitted.
